# Comment on Surendran et al. The Missed Opportunity of Patient-Centered Medical Homes to Thrive in an Asian Context. *Int. J. Environ. Res. Public Health* 2021, *18*, 1817

**DOI:** 10.3390/ijerph19084683

**Published:** 2022-04-13

**Authors:** Sharon Ngoh, Wern Ee Tang, Edwin Chng, Phui-Nah Chong

**Affiliations:** 1 Parkway Shenton Pte Ltd., 20 Bendemeer Road #05-04, Singapore 339914, Singapore; edwin.chng@parkwayshenton.com.sg; 2 National Healthcare Group Polyclinics, 3 Fusionopolis Link, Nexus@One-North, #05-10, Singapore 138543, Singapore; phui_nah_chong@nhgp.com.sg

We have noted the views expressed by the authors of the article “The Missed Opportunity of Patient-Centered Medical Homes (PCMH) to Thrive in an Asian Context” by Surendran et al., which was published in the *International Journal of Environmental Research and Public Health* (2021; 18: 1817) [1]. However, not all of the Family Medicine Clinics (FMCs) (the local version of the PCMH) in Singapore participated in the study. The Ang Mo Kio Family Medicine Clinic (AMK FMC) was one such PCMH that was not represented in the study. Here, we share our experience of the successful implementation of this PCMH in Singapore.

The Singapore population is ageing rapidly, with the average life expectancy of Singaporeans now at 84.9 years; many people are spending a considerable proportion of their later years in ill health [2]. The FMC model is an important public–private collaboration which has, at its core, family physicians skilled in chronic disease management, supported by an ancillary staff. This initiative aims to address the health needs of the rapidly ageing population by providing effective chronic disease management in primary care [3].

AMK FMC is a private–public collaboration between the Parkway Shenton Medical Group (privately run healthcare organization) and the National Healthcare Group (NHG) (publicly funded healthcare organization) that was set up in 2013. Both Parkway Shenton and NHG Polyclinics worked closely together and were jointly involved in decision-making processes during the initial setup and establishment of AMK FMC. The vision shared by both stakeholders is that of a primary care clinic helmed by a competent healthcare team that provides personalised and continuous care to patients. Experienced family physicians trained in chronic disease management lead the AMK FMC clinic team. During the start-up phase, NHG Polyclinics provided advice on organising a multidisciplinary team, training nurses and operations staff, as well as laboratory and pharmacy support services. To this end, Parkway Shenton and NHG invested in the hiring and training of clinic staff, and the provision of adequate operational support during the establishment of the clinic and its continued operations. An engaged leadership and the adequate staffing of trained members to provide quality team-based care have been described to be crucial elements of a PCMH model [4,5].

AMK FMC has been in operation for 8 years. At the start of operation in 2013, the clinic was led by two family physicians supported by two staff nurses, two patient service officers, one laboratory technician, one pharmacist, one pharmacy technician, one dietician and one cleaner. Over the years, its staff strength has grown steadily, to thirty in 2021 (four family physicians, five staff nurses, six patient service officers, one care facilitator, two laboratory technicians, two phlebotomists, one laboratory assistant, five pharmacy technicians, one triage staff member, one clinic manager, one operations manager and one cleaner). In addition to doctor consultations, AMK FMC provides onsite nurse counselling services, diabetic foot and diabetic retinal screening, a laboratory and pharmacy-dispensing services to patients. The clinic provides good continuity of care for patients with chronic conditions, which has resulted in building good doctor–patient relationships.

Studies have shown that patients’ satisfaction rates are inversely related to the waiting time to see a doctor at the clinic [6,7]. An acceptable waiting time may vary between countries and culture, although it has been reported that patients are less likely to be dissatisfied if the wait time is kept to 30–40 min [8,9]. AMK FMC has maintained a 50th percentile for wait time of 6.00–10.85 min (data obtained from clinic’s internal audit from financial year [FY] 2020 defined as April 2020 to March 2021).

Through the efforts of a care facilitator hired by Parkway Shenton and the NHG Polyclinics’ team, a total of 8131 patients have transferred their care from Ang Mo Kio Polyclinic to the AMK FMC team from 2013 to the present. The quality of care at the AMK FMC has been evaluated using both process and clinical outcome indicators. Examples of process indicators are the percentage of diabetic patients with at least six-monthly testing for HbA1c and the percentage of patients with diabetes having ≥2 blood pressure measurements annually. The clinical outcome indicators used included the percentage of patients (≤75 years old) with HbA1c < 7%. AMK FMC’s diabetes process and clinical outcome indicators from FY2018 to FY2020 are shown in Table 1.

Process and clinical outcomes indicators are monitored by the clinic management and reviewed regularly by a Clinical Governance Committee which has representatives from both Parkway Shenton and NHG Polyclinics. AMK FMC’s performance, as measured by process and clinical outcome indicators such as those shown in Table 1, is comparable to that of Singapore Health Services (SingHealth), which is the largest healthcare cluster in Singapore [10]. Lim et al. reported that 81% of their diabetic patients have ≥2 HBA1c tests performed annually, 93.2% of them have ≥2 blood pressure measurements annually and 52.7% met the target of HbA1c < 7% [10].

Surendran et al. mentioned that some FMCs were less accessible and visible to the community. One of the other key factors to AMK FMC’s success thus far is its close proximity to the referring polyclinic, which allowed for easy access of diagnostic and imaging services not available at the former but present in the latter when needed. In addition, most of the referred patients lived in the vicinity of the polyclinic. Hence, the close proximity of AMK FMC meant that they did not have to travel too far to the facility. This eased the initial fear of change in care provider: elderly patients value geographical familiarity.

We acknowledge that AMK FMC shares similar challenges raised by the article. These include patients exhausting the quantum of their yearly subsidies or losing eligibility of their Community Health Assist Scheme (CHAS) subsidies, which are government grants given to help eligible patients with the cost of their medical treatment tiered to their monthly individual household incomes. Furthermore, there is also a limit on the amount given to each patient according to their medical conditions. Despite this limitation, AMK FMC has continued to receive excellent service ratings from patients through the years, and morale amongst clinic staff remain high. These have contributed to AMK FMC’s ability to continue to thrive in the 5 years post-handover. In addition, AMK FMC has been keeping the laboratory investigation and drug costs comparable with the polyclinics so that care at the FMC is more affordable. 

Since the handover of AMK FMC to Parkway Shenton by NHG in 2016, Parkway Shenton has been running the AMK FMC independently. It continues to work with NHG Polyclinics as a collaborative partner. Surendran S. et al. highlighted that setting aside structured time for meetings for reflection and sense-making should be enforced in order to build trust and relationship. In the case of AMK FMC, there are regular clinical governance meetings and close collaborations between Parkway Shenton and NHG Polyclinics that adhere to joint decision-making processes. As recommended by Kassai et al., regulation by the government and other stakeholders is needed for a public–private collaboration, so as to ensure that the private providers keep up with the public sector in terms of quality, access and costs of care [11]. 

Long-term financial sustainability of the clinic is a key concern. AMK FMC has been a financially viable clinic thus far as a result of stern commitment from both Parkway Shenton and NHG. This includes support in the form of transferring patients by NHG Polyclinics and the provision of medications at subsidized cost to patients. There are also plans in the pipeline for AMK FMC to collaborate with other public health institutions for the right-siting of suitable patients to increase the patient pool. In 2018, the Primary Care Network (PCN) was launched by the Ministry of Health, with a vision to provide holistic team-based care to patients with chronic conditions [12]. General practitioners who choose to participate in the PCN will receive grants from the MOH to support their daily clinical operations as part of the network’s funding initiative. For example, subventions are given for chronic disease management ancillary services such as nurse counselling and diabetic foot and eye screening [13]. Doctors who take care of patients with complex chronic medical conditions will also receive additional resources [13]. AMK FMC is part of the Parkway Shenton PCN, and these governmental schemes have helped the organisation to remain financially stable.

Implementation of a model such as AMK FMC is feasible and offers many opportunities for public and private providers to build on each other’s strengths and cooperate in areas such as policy development and practice transformation [14]. We believe that the key ingredients to a successful FMC model are:

(a) Stakeholders with a shared vision and collective commitment;
(b) Well-trained multidisciplinary team of healthcare providers offering a comprehensive range of patient services at a single location;
(c) Close-working partnership between government-funded polyclinics and FMC with dedicated teams working together in planning the service as well as in resolving issues;
(d) Health financing schemes such as governmental grants for the management of patients with chronic diseases

The FMC model has much potential to contribute to the quadruple aims of improving [4,15]:

1. Health of patients through better health outcomes and quality of life;
2. Patient experience through better care coordination and accessibility;
3. Provider experience through work satisfaction and engaged leadership;
4. Reducing costs through better affordability, lowered hospitalization rates and healthcare utilization in the long term.

As of now, AMK FMC continues to function well as a PCMH in Singapore, and would like to posit that with the appropriate strategies and commitment from relevant like-minded stakeholders, patient-centred medical homes can thrive in Singapore and the rest of Asia.

## Figures and Tables

**Table 1:** AMK FMC diabetes process and clinical outcome indicators.

**Process Indicators**	**FY2018**	**FY2019**	**FY2020**
Percentage of diabetic patients with ≥2 testing for HbA1c per year	98.2%	90.2%	90%
Percentage of diabetic patients with ≥2 blood pressure measurements taken per year	99.7%	97.5%	98.2%
**Clinical Outcome Indicator**	**FY2018**	**FY2019**	**FY2020**
Percentage of diabetic patients (≤75 years) with HbA1c ≤ 7%	57.5%	54%	56.7%

FY2018: April 2018–March 2019; FY2019: April 2019–March 2020; FY2020: April 2020–March 2021.

## Data Availability

The data presented in this study are not publicly available due to data policy of Parkway Shenton. Readers can write to the corresponding authors if they wish to gain access to the data upon reasonable request.
